# Commentary: The effect of acupuncture on blood glucose control in patients with type 2 diabetes: a systematic review and meta-analysis of randomized controlled trials

**DOI:** 10.3389/fendo.2025.1680556

**Published:** 2025-09-04

**Authors:** Yunpeng Xu, Qian Liu, Boyan Ma, Hongbo Jiang, Jian Liu

**Affiliations:** ^1^ The First Clinical Medical College of Lanzhou University, Lanzhou, China; ^2^ The Second Clinical Medical College of Lanzhou University, Lanzhou, China; ^3^ Gansu Province Maternity and Child-Care Hospital (Gansu Provincial Central Hospital), Lanzhou, China

**Keywords:** diabetes mellitus, type 2, acupuncture therapy, systematic reviews, meta-analysis, evidence-based medicine

## Introduction

We read with great interest the recent systematic review and meta-analysis by Si et al. (1), published in Frontiers in Endocrinology, titled “The effect of acupuncture on blood glucose control in patients with type 2 diabetes”. This study included 21 randomized controlled trials and comprehensively evaluated the effects of acupuncture on multiple clinical metabolic outcomes in patients with type 2 diabetes (T2DM), including blood glucose, lipid profiles, body weight, and insulin resistance. The authors also performed subgroup and sensitivity analyses to explore potential sources of heterogeneity, which enhances the clinical relevance and applicability of the findings. We highly commend the authors for their valuable contribution to this important area. However, in the spirit of academic rigor, we would like to highlight several methodological concerns that may affect the interpretation and generalizability of the results. We respectfully offer the following points for consideration and discussion by the authors and readers.

## Inconsistency between included studies and eligibility criteria

This study aimed to systematically evaluate the effects of acupuncture on clinical metabolic indicators in patients with type 2 diabetes (T2DM), and explicitly stated that “diagnosed with T2DM were eligible for inclusion.” However, we found that several included studies did not meet this criterion: the study by Luo et al. (2) involved patients with impaired glucose tolerance, those by Li et al. (3) and Xue et al. (4) included individuals with prediabetes, and the study by Sebayang RG et al. (5) focused on obese participants. In total, 4 out of the 21 included studies (19%) failed to align with the defined T2DM diagnostic standard. This selection bias is particularly prominent in certain secondary outcomes. For example, in Supplementary Figure 1 (E) Forest plot BMI, studies involving non-T2DM populations accounted for 42.8% of the total weight, which may substantially affect the representativeness and interpretability of the findings.

## Insufficient coverage of chinese literature databases

As this review focuses on acupuncture for type 2 diabetes—a topic deeply rooted in traditional Chinese medicine—it falls within the scope of a typical TCM-related systematic review. Given that a large proportion of acupuncture studies are published in Chinese-language journals, with 76% of the included studies authored by Chinese researchers and 57% published in Chinese, comprehensive coverage of Chinese biomedical databases is essential to ensure thorough literature retrieval. However, the authors searched only CNKI and Wanfang databases, without including the Chinese Biomedical Literature Database (CBM) or VIP. According to recommendations by Sichuan University and the Cochrane Centre in the UK, CBM is considered the primary database for Chinese biomedical research in systematic reviews, while CNKI, Wanfang, and VIP are suggested as supplementary sources (6). The omission of key databases may have led to the exclusion of relevant high-quality studies, potentially compromising the completeness and representativeness of the review findings.

## Unit-of-analysis error

This study presents a clear unit-of-analysis error. In meta-analyses, when multiple intervention groups share a common control group, it is essential to avoid duplicating the control data, as this compromises statistical independence, inflates precision, narrows confidence intervals, and increases the risk of false-positive findings (7). For instance, the study by Jiang et al. (8) included three groups: wrist-ankle acupuncture, body acupuncture, and conventional treatment. However, this review treated the comparisons “wrist-ankle acupuncture vs. control” and “body acupuncture vs. control” as two independent analyses, thereby using the same control group twice. A similar issue was found in the study by Wang et al. (9) Additionally, some outcomes—such as PCV, whole blood viscosity, and FIB in Supplementary Figure 1—were based on a single study, further limiting the robustness of the results. According to the Cochrane Handbook, this bias can be corrected by combining intervention groups (recommended), selecting only one comparison, splitting the control group, or using network meta-analysis methods (7).

## Incorrect calculation of standard deviation for pre-post differences

In this review, most outcomes were analyzed based on pre-post differences (i.e., post-treatment minus pre-treatment values). However, the method used to calculate the standard deviation (SD) of these changes appears to be flawed. Based on the available data, it seems the authors assumed a correlation coefficient (r) of zero between pre- and post-treatment measures, thereby ignoring the within-subject correlation inherent in paired designs. As noted in the Cochrane Handbook, assuming r = 0 can lead to substantial overestimation of SD_change, underestimation of effect size, and reduced statistical power (10). A more appropriate approach involves estimating SD_change from the known baseline and final SDs using a reasonable assumption or borrowed r value (e.g., r = 0.5 or 0.7), followed by sensitivity analyses to assess the impact of varying r. This methodological error may have influenced the accuracy of several effect estimates (10). The recommended formula is as follows:


$$SD_{change}=\sqrt{SD_{baseline}^{2}+SD_{final}^{2}-2r\cdot SD_{baseline}\cdot SD_{final}}$$

## Misclassification of intervention groups

We observed a misclassification issue in the handling of intervention arms from the study by Wang et al. (9) In the original trial, only groups A and B did not receive acupuncture, whereas groups C, D, and E were all treated with some form of acupuncture. However, in the meta-analysis, group E was categorized as the acupuncture intervention group, while the remaining four groups were treated as controls. This classification contradicts the actual intervention allocation and introduces potential confounding bias, which may distort effect estimates and undermine the interpretability and validity of the study’s conclusions.

## Discussion

While this systematic review provides valuable insight into the potential role of acupuncture in glycemic control for patients with T2DM, the aforementioned methodological issues may compromise the validity and generalizability of its findings. We recommend that future studies clarify inclusion criteria more rigorously, expand database coverage—especially of Chinese literature—and adopt Cochrane-recommended approaches to strengthen analytical strategies. We look forward to further investigations that, under robust study design and supported by high-quality evidence, will more comprehensively assess and validate the clinical potential of acupuncture in the management of diabetes.
